# Candidate gene-environment interactions in breast cancer

**DOI:** 10.1186/s12916-014-0195-1

**Published:** 2014-10-17

**Authors:** Olivia Fletcher, Frank Dudbridge

**Affiliations:** Breakthrough Breast Cancer Research Centre, The Institute of Cancer Research, London, SW3 6JB UK; Division of Breast Cancer Research, The Institute of Cancer Research, London, SW3 6JB UK; Department of Non-communicable Disease Epidemiology, London School of Hygiene and Tropical Medicine, London, WC1E 7HT UK

**Keywords:** Gene-environment interaction, Breast cancer, Single nucleotide polymorphism

## Abstract

Gene-environment interactions have the potential to shed light on biological processes leading to disease, identify individuals for whom risk factors are most relevant, and improve the accuracy of epidemiological risk models. We review the progress that has been made in investigating gene-environment interactions in the field of breast cancer. Although several large-scale analyses have been carried out, only a few significant interactions have been reported. One of these, an interaction between *CASP8*-rs1045485 and alcohol consumption has been replicated, but others have not, including *LSP1*- rs3817198 and parity, and 1p11.2-rs11249433 and ever being parous. False positive interactions may arise if the gene and environment are correlated and the causal variant is less frequent than the tag SNP. We conclude that while much progress has been made in this area it is still too soon to tell whether gene-environment interactions will fulfil their promise. Before we can make this assessment we will need to replicate (or refute) the reported interactions, identify the causal variants that underlie tag-SNP associations and validate the next generation of epidemiological risk models.

## Background

Epidemiological studies have provided consistent evidence of associations between environmental (predominantly lifestyle and reproductive) factors and subsequent risk of breast cancer (BC). More recently, genome-wide association studies (GWAS) have identified more than 70 single nucleotide polymorphisms (SNPs) that influence breast cancer risk [1]. Detecting a gene-environment (GxE) interaction between a SNP and an environmental risk factor has the potential to shed light on the biological process leading to disease, identify women for whom these risk factors are most relevant, and improve the accuracy of epidemiological risk models [2]. A comprehensive review summarising the rationale for and the challenges of studying GxE interactions advocated a range of measures including supporting new and larger prospective studies, the reporting of stratified analyses as supplementary material and pre-planned analyses coordinated across multiple studies [2]. In this commentary we review progress in investigating GxE interactions in the field of BC. We define GxE interaction as the modification of the effect of a genetic risk factor by an environmental factor, assessed statistically by testing the effects of gene and environment for departure from additivity, on an appropriate scale (usually the log or logit in disease studies). We focus on GxE interactions between common SNPs and established risk factors for BC (Table 1), discuss the implications of testing marker SNPs rather than the underlying causal variants that they tag and consider whether GxE studies have fulfilled their potential for illuminating disease processes or predicting risk.

**Table 1:** **Established risk factors assessed in GxE interaction studies**

**Established risk factor**	**Travis** ***et al*** **. [** 3 **]**	**Milne** ***et al*** **. [** 4 **]**	**Campa** ***et al*** **. [** 6 **]**	**Nickels** ***et al*** **. [** 5 **]**	**Barrdahl** ***et al*** **. [** 7 **]**	**Schoeps** ***et al*** **. [** 12 **]**
Age at menarche (years)	X	X	X	X	X	X
Age at first birth (years)	X	X		X^a^	X	
Parous (% and/or number of live births)	X	X	X	X^a^	X	X
Breast fed (% in parous women)	X			X		
Menopausal status (% post-menopausal)	X					
Age at natural menopause (years)	X		X		X	
Use of oral contraceptives (yes/no or duration)				X	X	X
Use of HRT (yes/no or duration)	X		X	X		X^b^
Body Mass Index (kg/m^2^)	X	X	X	X	X	X^c^
Height (m)	X		X	X	X	X
Alcohol consumption (g per day)	X		X	X	X	X
Smoking (pack-years)			X	X	X	
Family history of BC			X		X	X
Physical activity (hours per week)				X		

^a^The ten established risk factors reported by Nickels *et al*. (Table 2) counts parity and age at first live birth as a single factor; ^b^the 10 established risk factors reported by Schoeps *et al*. (Table 2) counts combined estrogen-progesterone and estrogen only post-menopausal hormone therapy as two factors; ^c^body mass index in pre- and post-menopausal women as two factors. BC, breast cancer; HRT, hormone replacement therapy.

### GxE interactions between previously reported SNPs and established risk factors for BC

The first large (that is, at least 5,000 cases and 5,000 controls) GxE study of this type was carried out within the Million Women Study [3]. In this analysis of 7,610 cases and 10,196 controls investigating potential GxE interactions between 12 SNPs and 10 established risk factors for BC there were no GxE interactions that were significant after adjusting for multiple testing. The most significant GxE interaction was between *CASP8*-rs1045485 and alcohol consumption (unadjusted *P* = 0.003). Since the publication of this report, there have been four further analyses of this type (Table 2), two from the Breast Cancer Association Consortium (BCAC) [4,5] and two from the Breast and Prostate Cancer Cohort Consortium (BPC3) [6,7]. Only one of these, the largest (23 SNPs in 34,793 cases and 41,099 controls) [5], reported statistically significant GxE interactions, namely between *LSP1*-rs3817198 and parity (number of live births), *CASP8*-rs1045485 and alcohol consumption (replicating the most significant finding in the Million Women study [3]) and 1p11.2-rs11249433 and ever being parous. However, none of these interactions was replicated in the largest BPC3 study (39 SNPs in 16,285 BC cases and 19,376 controls [7]). A meta-analysis of the BCAC and BPC3 data suggested a possible interaction between *SLC4A7*-rs4973768 and smoking status but replication of this result has not yet been attempted.

**Table 2:** **Details of GxE interaction studies comprising at least 5,000 cases and 5,000 controls**

**Reference**	**Study**	**SNPs**	**ERFs**	**Cases**	**Controls**	**Strongest interaction**	**Interaction effect size**	**Unadjusted** ***P***
Travis *et al*. [3]	Million women	12	10	7,610	10,196	*CASP8*-rs1045485; alcohol consumption^a^	1.24	0.003
Milne *et al*. [4]	BCAC (1)	12	4	26,349	32,208	*LSP1*- rs3817198; parity	1.05	0.002
Campa *et al*. [6]	BPC3 (1)	17	9	8,576	11,892	5p12-rs10941679; use of estrogen only HRT	1.22	0.0072
Nickels *et al*. [5]	BCAC (2)	23	10	34,793	41,099	*LSP1*- rs3817198; parity	1.06	2.4 × 10^−6^
						1p11.2-rs11249433; ever parous	1.16	5.3 × 10^−5^
						*CASP8*-rs17468277; alcohol consumption^a^	1.59	3.1 × 10^−4^
Barrdahl *et al*. [7]	BPC3 (2)	39	10	16,285	19,376	6q25-rs2046210; alcohol consumption^a^	1.11	0.002
Schoeps *et al*. [12]	BCAC	71,527	10	34,475	34,786	21q22.12-rs10483028 and rs2242714; postmenopausal BMI	0.84	3.2 × 10^−5^

^a^Alcohol consumption was defined as per additional 10 g in Travis *et al*. and Barrdahl *et al*. and as < or ≥20 g/per day by Nickels *et al*. BCAC, Breast Cancer Association Consortium; BMI, body mass index; BPC3, Breast and Prostate Cancer Cohort Consortium; ERFs, established risk factors for breast cancer; HRT, hormone replacement therapy.

The Shanghai Breast Cancer Genetics Study tested for interactions using a risk score formed as the weighted sum of genotypes from 10 SNPs [8]. This would improve the power to detect a risk factor that has interactions with numerous SNPs, when there is insufficient power for the individual interactions. Although this study found no interactions with the risk score, this approach holds promise for identifying interacting risk factors in limited sample sizes.

### Identification of novel risk SNPs through GxE interactions

SNPs with strong interaction effects may only be detectable when analysing gene and environment together, so they are missed by studies that consider SNPs in isolation. Methods that model and test the main and interaction effects of gene and environment jointly [9], or exploit the power of a case-only design while retaining robustness to possible gene environment dependence [10,11] have been developed for these purposes. Recently, several of these methods were applied to 71,527 SNPs with suggestive association with BC [12]. Interactions were identified between two SNPs on 21q22.12 (rs10483028 and rs2242714) and adult body mass index (BMI), and one in *ARID1B* (rs12197388) with age at menarche and with parity. rs12197388 was only significant in the joint test of main and interaction effects, and the interaction term was not significant but the two SNPs on 21q22.12 were detected via their interactions, and further studies of this nature may discover more interactions using these novel methods.

### Using tag-SNPs as proxies for an underlying causal variant

The GxE studies described above have relied on using marker SNPs, predominantly identified through GWAS, as proxies for the underlying causal variants. This usually leads to a loss in power to detect interactions [13]. However, if gene and environment are dependent, a marker SNP can show an interaction even if there is no interaction at the causal variant [14]. These ‘spurious interactions’ tend to arise when the causal variant is rare in comparison to the marker. This may not often be the case, but it nevertheless warrants caution when reporting GxE interactions. We recently studied a marker SNP (rs10235235) associated with a reduction in urinary levels of an estrogen metabolite [15]. In 47,346 cases and 47,569 controls in the Collaborative Oncological Gene-environment Study (COGS) [1,16] this SNP showed (1) association with BC risk, (2) association with age at menarche in controls (but not cases) and (3) an interaction in which age at menarche modified the effect of rs10235235 on BC risk. In this example of a GxE interaction, therefore, the genetic risk factor (rs10235235) is dependent on the environmental risk factor (age at menarche), which could lead to a false positive [14]. Of the interactions reported to date, gene-environment dependence has been observed between *LSP1*-rs3817198 and parity and 21q22.12-rs10483208/rs2242714 and BMI. In cases such as these, an interaction can only be definitively established when all variation in the associated regions has been identified and tested.

## Conclusions

Several of the recommendations made by Hunter in 2005 [2] have been pursued: large new prospective studies continue to be supported (for example the Breakthrough Generations study, a long-term cohort study focused on BC has recruited 112,049 women over the period 2003 to 2011 [17]), consortia of case–control (BCAC) and cohort studies (BPC3) have coordinated their efforts for analyses of data from >70,000 women and the results of stratified analyses have been conscientiously reported in supplementary tables [5,7]. However, one of the lessons of the first generation of BC GWAS [18-20] was that the per-allele disease odds ratios (ORs) associated with individual tag-SNPs were much smaller than hypothesised (1.07 to 1.26). Results from the first generation of GxE analyses suggest that the same may be true for interactions, with the reported interaction ORs ranging from 1.06 to 1.59. If marginal ORs of 1.07 to 1.26 require scans of several thousand cases and several thousand controls then, depending on the number of GxE interactions being tested, only GxE studies that include tens of thousands of cases and controls will have the power required to detect interactions. It is hardly a coincidence that the first study to report statistically significant GxE interactions was the first study of this order of magnitude [5]. Of the three significant interactions reported by Nickels and colleagues there is replication only for *CASP8*-rs1045485 and alcohol consumption. It is currently too soon to tell whether GxE interactions will shed light on disease processes and improve the accuracy of epidemiological risk models. Before we can make this assessment we will need to replicate or refute the reported interactions, identify the causal variants that underlie tag-SNP associations and validate the next generation of epidemiological risk models.

## Abbreviations

BC: breast cancer
BCAC: the Breast Cancer Association Consortium
BMI: body mass index
BPC3: the Breast and Prostate Cancer Cohort Consortium
COGS: Collaborative Oncological Gene-environment Study
ERF: established risk factor
GWAS: genome-wide association study
GxE interaction: gene-environment interaction
HRT: hormone replacement therapy
OR: odds ratio
SNP: single nucleotide polymorphism
